# Exploration of Policy Makers’ Views on the Implementation of the Framework Convention on Tobacco Control in the Gambia: A Qualitative Study

**DOI:** 10.1093/ntr/ntz003

**Published:** 2019-01-09

**Authors:** Isatou K Jallow, John Britton, Tessa Langley

**Affiliations:** 1 UK Centre for Tobacco and Alcohol Studies, Division of Epidemiology and Public Health, University of Nottingham, Nottingham, UK; 2 National Public Health Laboratories, Ministry of Health & Social Welfare (MoH&SW), The Gambia

## Abstract

**Background:**

The World Health Organization’s Framework Convention on Tobacco Control (FCTC) is the first international health treaty and has now been ratified by 181 countries. However, there are concerns that in many countries, particularly in sub-Saharan African countries, FCTC legislations and implementation are weak. In this study, we report a qualitative study undertaken to assess policy makers’ awareness of the FCTC and national tobacco control policies, and assessed the achievements and challenges to the implementation of the FCTC in the Gambia.

**Methods:**

The study involved semi-structured one-to-one interviews with 28 members of the National Tobacco Control Committee in the Gambia, which is responsible for formulating tobacco control policies and making recommendations for tobacco control. We used the Framework method and NVivo11 software for data analysis.

**Results:**

Our findings demonstrate that the Gambia has made modest progress in tobacco control before and since ratification of the FCTC, particularly in the areas of policy formulation, bans on tobacco advertising and promotion, smoke-free laws, and tobacco taxation. Although several pieces of tobacco control legislation exist, enforcement and implementation remain a major challenge. We found that policy makers’ awareness of polices covered in the FCTC was limited.

**Conclusion:**

Our findings highlight several challenges to the FCTC implementation and the need to step up efforts that will help to accomplish the obligations of the FCTC. To achieve the obligations of the FCTC, the Gambia should develop specific public awareness interventions, establish cessation services, mobilize adequate resources for tobacco control and strengthen tobacco surveillance and research.

## Introduction

The World Health Organization’s Framework Convention on Tobacco Control (WHO FCTC), the first international health treaty, entered into force in 2005. It has since become one of the most widely and rapidly embraced treaties in the history of the United Nations, with 181 Parties to date.^1^ The WHO FCTC was developed in response to the globalization of the tobacco epidemic and is an evidence-based treaty that reaffirms the right of all people to the highest standard of health. It presents a unique opportunity to reduce the global burden of tobacco-related mortality and morbidity. The FCTC is particularly important in developing countries, such as those in sub-Saharan Africa, where tobacco smoking is on the rise.^2,3^ The FCTC requires Parties to implement all tobacco control measures outlined in the convention. (See Supplementary File 1 for details of the FCTC articles and measures). For the FCTC measures to be successful, it is essential that ratification is followed by implementation;^4^ however, there are concerns that in many countries legislation and implementation of FCTC are weak.^4^ In developing countries, particularly in many sub-Saharan African countries, data on the progress of implementation are limited.

Tobacco control has been a focal point for the Ministry of Health & Social Welfare in the Gambia since 2002 long before ratification of the FCTC. A detailed overview of essential point of legislation and tobacco control policy documents is outline in Table 1. In 2012, the Convention Secretariat conducted a needs assessment for implementation of the WHO FCTC in the Gambia and found that the Gambia has not met several obligations under the Convention, and progress on implementation has been slow.^5^ Tobacco control activity has included the formation of a National Tobacco Control Committee in July 2012. The Committee comprises partners and stakeholders from government ministries, civil society organizations, and nongovernmental organizations, and has responsibility for formulating tobacco control policies and making recommendations for tobacco control. However, the extent of progress in the implementation of tobacco control policies in the Gambia remains unknown. This study reports the findings of a qualitative study undertaken to assess policy makers’ awareness of the FCTC and national tobacco control policies, and assess the achievements in and challenges to the implementation of the FCTC.

**Table 1.** Details of the Essential Points of Legislation and Existing Policy Documents in the Gambia

Time points	Legislation and policy documents	Details
1998	Prohibition of Smoking (Public Places) Act	Comprehensively bans smoking in any enclosed (?) public place, workplace, hospital, public vehicle, or Government premises
2003	Ban on Tobacco Advertisements Act	Ban on advertisement or promotion of a tobacco product in any form
2007	Framework Convention on Tobacco Control (FCTC)	Ratification of the FCTC and entry into force
2009	Health Warning Directives	Heath warnings that describe the harmful effect of tobacco use must occupy 30% of the principal display areas on both sides of cigarette packs and include a Sold in The Gambia label
2012	Needs Assessment	Convention Secretariat Needs Assessment for implementation of the WHO FCTC in the Gambia
2012	National Tobacco Control Committee	Formation of a multi-sectorial working group, which comprises partners or stakeholders from government ministries, civil society organizations and nongovernmental organizations, and has responsibility for formulating tobacco control policies and making recommendations for tobacco control
2013	Tax increase policy	Three year tobacco tax policy was developed, which was implemented 2014–2016
2013	National Tobacco Control Policy and Action Plan	Outlines the strategic direction that will be pursued in the control of tobacco in the Gambia between 2013 and 2018
2016	Tobacco Control Act 2016	Aims to control the demand and supply of tobacco-related products, implement the WHO FCTC
2016	National Clinical Guideline for Cessation Services	Launching of a 3-year national tobacco cessation clinical guideline
2016	Illicit Trade Protocol	Accession to the Protocol on Illicit Trade in Tobacco Products

## Methods

### Study Participants and Design

The study involved qualitative one-to-one interviews with members of the National Tobacco Control Committee (see Introduction section). All 35 members of the committee were contacted by telephone to book an appointment for the interview, and written consent obtained before the start of the interview. Face-to-face interviews were carried out with all consenting members using a semi-structured interview guide adapted from a similar study conducted in Ghana.^6^ All interviews were conducted in English, which is the main official language. However, most Gambians speak various local languages, and three participants sometimes spoke in local languages during the interviews. All the local languages spoken during the interviews were languages the interviewee understood. The interview guide covered members’ perceptions of current tobacco use in the Gambia, awareness of current tobacco policies, and implementation of various aspects of the WHO FCTC. The interview also covered challenges, achievements, and recommendations for implementing specific tobacco control measures. (See Supplementary File 2 for details of the interview questionnaire). All interviews were audio-recorded and transcribed verbatim. In instances where participants spoke in local languages, this was directly translated in English during transcribing by the led researcher. Data collection was conducted between June and September 2016.

### Analysis

We used the Framework method, which is commonly used to analyze semi-structured interviews.^7,8^ The data were analyzed using NVivo software version 11 (QSR International, Australia). The first stage of the analysis involved preparing and organizing the data by listening back to all the audio-recorded interviews and reading all the interview transcripts to become familiarized with the data. After familiarization with the data, the first few transcripts were reduced into themes and subthemes through a process of coding by the lead researcher. For quality control purposes, a random sample of nine transcripts was double coded by two further researchers to confirm the consistency of coding. To reach consensus on all the initial themes and subthemes and to resolve any coding discrepancies, all authors met to discuss the codes assigned to each transcripts and all coded sections of each of the transcripts, in terms of why it had been interpreted as meaningful and what it told us about participants’ views on tobacco control polices in the Gambia.

After discussions identified themes and subthemes, these were given unique codes and an initial thematic node hierarchy was set up for all identified themes. The lead researcher then coded the remaining transcripts using the initial Framework, taking note of any new codes or themes that did not fit into the initial themes and subthemes. All researchers met again and, following discussions, the initial Framework was revised to incorporate new codes, refined existing codes, and grouped related codes. The process of refining, applying, and refining the analytical Framework was repeated until no new codes were generated. A thematic node hierarchy was set up for all identified themes and subthemes, and the final analytical Framework was applied to all transcripts in NVivo. Once all the transcripts were coded using the Framework, the data were summarized in a matrix for each of the main themes and subthemes using the framework matrices in NVivo. (See Supplementary File 3 for more details of the Framework method analysis used and an illustrative example in NVivo). The main themes were allocated to each row on the chart and each transcript was assigned to a specific column. This process allows for rearranging the data according to the appropriate part of the thematic Framework to which they relate and the process of charting also ensures that all coded data and context are included in the charts. Finally, the chart matrices were used to identify the differences and similarities across transcripts and within themes, to explore relationships and association between the themes and concepts.

### Ethics Approval

This study was approved by the Gambia Government and Medical Research Council Joint Ethics Committee (SCC 1468v2), and the Faculty of Medicine and Health Sciences Research Ethics Committee, University of Nottingham (OVS24022016 SoM EPH).

## Results

Of the 35 members of the National Tobacco Control Committee, one declined to participate, six agreed but were unavailable for interview during the study period, and 28 were interviewed. The interviews lasted between 29 and 70 minutes. The interview time varied according to participant’s roles and responsibilities and their knowledge of tobacco control in the Gambia. Participants included individuals from all the ministries of the government, and from nongovernmental organizations, civil society organizations, research institutions, private institutions, parastatals, WHO, security services, and media institutions. The sociodemographic characteristics of the participants are presented in Table 2. The mean age of participants was 43.6 years and the age range was 28–65 years. The majority of those interviewed were male (89.2%), never-smokers (67.8%), and worked at government ministries and agencies (75%).

**Table 2.** Participant Sociodemographic Characteristics

Characteristics	Total (*N* = 28)	%
Age (years)		
28–38	11	39.2
39–49	9	32.1
50+	8	28.5
Gender		
Male	25	89.2
Female	3	10.7
Smoking status		
Never-smokers	19	67.8
Ever-smokers	9	32.2
Representing institutions		
Government ministries and agencies	21	75.0
Others	7	25.0

### Themes and Subthemes

Eight main themes were identified from the subthemes. The themes and subthemes are summarized in Table 3, and participants’ quotes are provided in italics.

**Table 3.** Themes and Subthemes Identified

Themes	Subthemes
Framework Convention on Tobacco Control (FCTC) and national polices	• Knowledge of FCTC
	• Awareness of national tobacco polices
	• Coverage of FCTC in national polices and legislation
Public smoking (smoke-free policy)	• Implementation
	• Lack of awareness of health risks and legislation
	• Weak enforcement
Tax and illicit trade	Achievements
	• Tax increase plan
	• Increasing revenue
	• Decreasing volume of importation
	Challenges
	• Price and affordability
	• Cheaper brands and products
	• Porous borders and country geography
	• Customs challenges
Mass media	• Need for public awareness
	• Enabling factors
	• Lack of resources
Advertisement and promotion	Achievements
	• Success in enforcement
	• High level of compliance
	Challenges
	• Indirect form of advertisement
	• Ongoing sponsorship
Access	• Sales to minors
	• Reasons for youth access
Stop smoking support	• Lack of stop smoking services
	• Use of traditional treatments
Packaging and labeling	• Warning labels currently in use
	• Language barriers and illiteracy
	• Need for pictorial warnings

### FCTC and National Tobacco Control Polices

Most participants were aware of and understood the current existing national tobacco polices. However, one consistent issue that emerged was that the current policies were inadequate and implementation was lacking. The majority of members were optimistic that when the new bill comes into law, implementation and enforcement of these laws will be much more effective. Despite their limited knowledge of the FCTC, most participants believed that policies that predated the current bill did not adequately address FCTC requirements.

*Yes there are several Acts and legislations dealing with smoking. There is a policy for public smoking ban and advertisement. However, these past Acts and the Bills are not effective, but this new draft bill if it is endorsed I think it will be effective. This new bill has an enforcement plan, which addresses the enforcement part.* **Participant 022**

### Smoke-Free Policy

Participants reported that despite the implementation of the Public Smoking Act in 1998, little had been achieved with the ban on public smoking. Participants identified lack of awareness of the health risks of smoking, limited knowledge of existing legislation, weak enforcement, minimal fines, and law enforcers’ smoking have contributed to the challenges of achieving the smoke-free public places.

*I think it’s still a challenge. Because of enforcement it’s the biggest challenge for this policy. We’ve seen even still now hospitals are not smoke free zones. Everywhere people can smoke anyhow they want and they can walk freely without any fine. Also most law enforcers like the police are smokers and you know what you see them openly smoking in the streets. I think we need to do more to educate everyone about this law.* **Participant 009**

The lack of success was also attributed to cultural factors such as the “Maslaha Syndrome” (socially accommodating negative habits or behaviors and trying to cover it up in order not to be blamed for reporting it).

*The “Maslaha” syndrome, contributes a lot to the problem of implementing the public smoking Act. Because if you want to address somebody or even report the person to the police for smoking in prohibited area, they will say “ah” this is a fellow human being or it’s a relative, “why should you do that?”, so people’s attitude is a problem.* **Participant 019**

### Tax and Illicit Trade

All participants said that the Gambia has achieved a lot in relation to tobacco taxation. Participants highlighted that the 3-year tax increase plan has increased the revenue collected from tobacco and that the Gambia has seen a decrease in the volume of tobacco imports. However, many believed that further increases in tax are needed as tobacco products are still cheap and affordable to many Gambians.

*There is achievement in terms of tobacco taxation, but we still need to increase the price because it’s still cheaper in the Gambia than many countries. Actually most of the sweets here cost D1, so it’s like cigarette is equal to a sweet, so it’s still cheap, kids can buy it … oh yes it’s very cheap.* **Participant 028**

Nearly all of the respondents were of the view that smuggling of tobacco products could potentially be occurring, due to the fact that the Gambia has several porous borders and that increased efforts are needed to monitor smuggling of tobacco products coming both into and out of the country.

*Smuggling and illicit trade are some of the concerns we have now. We have been consistently increasing the taxes on the product from 2013 to date. The fact that we have several porous borders means that we need to strengthen our border controls now. We also need a very good tracking and tracing system.* **Participant 017**

They said that a tag and trace system currently exists, which involves labeling all cigarette packs with “Sold in The Gambia,” but that this alone is inadequate for tracking illicit tobacco products. Most were of the view that customs officers at border posts need to be trained and motivated by rewards and incentives for identifying illicit products. Participants highlighted, however, that resources for dealing with this problem are very low and that this posed a problem.

### Public Awareness

Mass-media campaigns, education, and communication were an area of tobacco control, which some of the respondents said, were ongoing in the Gambia but the majority said not much has been achieved.

*Well there are some forms of sensitization going on in radios but this is not adequate, therefore a lot still needs to be done to educate people about the dangers of smoking.* **Participant 009**

The need for more public awareness was mentioned by nearly all interviewees, and they also identified that several opportunities exist to improve this through school education, radios, and using existing community structures. Furthermore, most participants highlighted that there are very limited resources available for tobacco control activities and particularly for public awareness campaigns.

*No it’s still a major challenge, but it’s a major challenge because there’s no continuity, because of lack of funds, because these things they are not free. What I think is required here is mass sensitization should be done in a different way to address all issues. That means we have to use the radio, we have to use the television, we have to use the newspapers, one to one, there have to be organizing workshops, you know especially in the communities, local people and in schools.* **Participant 005**

### Advertising and Promotion

Respondents were of the opinion that advertisement and promotion were one of the areas of success of tobacco control in the Gambia. Furthermore, most attributed this success to enforcement of the Ban on Tobacco Advertisement Act of 2005. However, many participants hoped that these gains can be further consolidated when the tobacco control Draft Bill is implemented.

*I think this is one area we’ve made progress. When this law entered into effect, you don’t see any billboard signs about cigarette smoking or advertising cigarettes and so on around. Before I do see in some newspapers, billboard and even in radio stations advertising tobacco. Now I don’t see or hear of any these advertisements going on.* **Participant 023**

Some raised concerns about some of the indirect forms of advertisement going on, such as the use of tobacco brand names and logos on vehicles and umbrellas and sponsorships.

*Yeah there are definitely less adverts currently happening. The only forms of adverts you see sometimes is the indirect advertisement, in some occasions you will see vehicles being painted in cigarette colours and umbrellas.* **Participant 009**

### Access

Respondents unanimously agreed that until now, very little had been achieved in preventing young and underage children accessing cigarettes. Many raised concerns that parents and adults sending young people to purchase cigarettes were a big problem in the Gambia. They said that although the current Draft Bill includes minimum age for the sale to, and by, minors, nothing is being done currently to check access to and use of tobacco products by minors.

*It’s still one of the biggest problems because in The Gambia especially parents and adults send children to buy cigarettes for them and some even go to the extent of asking children to lighten the cigarettes for them. Having Age limit is important it should not be made easy for a child to access cigarette.* **Participant 014**

In addition, parents’ and retailers’ lack of knowledge about the harmful effects of smoking and exposure to secondhand smoke was highlighted as a major barrier.

*You know many parents and even the shopkeepers do not know that it’s illegal to send or sell cigarette to children under the age of 18 years. In fact some parents and even retailers don’t even know the harmful effects of smoking and what the health effects of exposing their kids to second-hand smoke.*

### Smoking Cessation Services

Many of the respondents were of the view that little has been done in providing support to reduce demand for tobacco use. In the absence of this service, many raised concern that most smokers will resort to using local traditional treatment methods that may be ineffective.

*This is a major challenge and currently there is no services provided for people who want to quit. That’s why some people go to the Marabous (traditional healers) to help them to quit smoking. I could fully remember when I visited one such Marabou. However you don’t know what he or she is giving you to use so this could be dangerous.* **Participant 011**

Many also praised the launching of the clinical guideline for cessation services by the Ministry of Health; however, many said that more efforts are needed if the Gambia is to achieve anything meaningful.

*Its one thing to sensitise people but it’s another thing also to have those avenues where you can help these people to quit. The launching of the national cessation guideline recently and training of healthcare workers in five clinics will definitely provide help for smokers to try and quit smoking.* **Participant 029**

### Packaging and Labeling

Most respondents thought there had been achievements in packaging and labeling of tobacco products, particularly cigarette packaging that has largely conformed to the current laws and requirements (health warning messages describing the harmful effects of tobacco use, covering 30% of the principal display of each unit of tobacco packaging and Sold in The Gambia label). However, they were also quick to say that more needs to be done, due to the fact that packages currently carry only written health warnings and that many Gambians may not be able to understand these messages. The use of graphic warning labels was recommended by all participants.

*Yes we have achieved something, but I think there are still some challenges, because the health warning on tobacco products especially cigarette packs in The Gambia have a written health warning, but the challenge there is most of the people in The Gambia cannot read and write. Most people and even smokers will not understand the words in the health warning. So we are advocating for pictorial warning, and that’s a challenge because that’s not captured yet.* **Participant 009**

## Discussion

This study provides the first data on the progress of implementation of the FCTC in the Gambia using qualitative methods. Our findings demonstrate that the Gambia has made modest progress in tobacco control before and since ratification of the FCTC, particularly in terms of bans on tobacco advertisement and promotion, smoke-free laws, and tobacco taxation. However, although several pieces of tobacco control legislation exist, enforcement and implementation remain a major challenge. We found that policy makers’ awareness of polices covered in the FCTC was limited. Furthermore, there was also a belief among policy makers that the general public’s awareness of existing tobacco control legislation was low.

Our findings are somewhat limited by the fact that our data were collected in 2016. However, apart from the passing of the Tobacco Control Act 2016 (which outlines key strategies for implementing tobacco control polices in the Gambia) into law, there has not been any major policy changes since we collected our data. Therefore, all our findings remain relevant to tobacco control in the Gambia. Furthermore, our study has a number of strengths and has provided valuable findings for a review of tobacco control efforts in the Gambia. One of the strengths of our study is that our findings are based on data from a purposive sample of members of the multi-sectoral committee, who are likely to have the best overview of the state of tobacco control in the Gambia. We also used the Framework method for analysis, which has the strength to produce credible and relevant findings.^8^

WHO has developed a simple Framework to guide and measure the implementation of specific provisions of the FCTC, known as MPOWER. These measures include Monitoring tobacco use (Article 20), Protecting people from exposure to secondhand smoke (Article 8), Offering help to quit (Article 4), Warning about the dangers of tobacco (Articles 11 and 12), Enforcing bans on tobacco advertising and promotion (Article 13), and Raising taxes on tobacco (Article 6).^9^ To achieve maximum benefit of the FCTC and the MPOWER measures, all polices in it need to be implemented as they are all complementary and synergistic to each other.^9^ The means to control the tobacco epidemic are therefore clear, and implementing these control measures is crucially important for African countries that are generally in the early stages of the tobacco epidemic model.^10,11^

Like many other developing countries,^12^ and in particular sub-Saharan African countries,^6,10^ the Gambia started developing and implementing some tobacco control polices that are covered by MPOWER before the ratification of the FCTC. Thus, the ratification provided an opportunity for the country to strengthen existing polices and set up a legal Framework for implementing tobacco control measures. Whereas some countries have made very substantial progress since ratification of the Treaty, many countries have found implementation to be rife with challenges.^13^ Implementing effective tobacco control policy is a major challenge for many governments particularly those in developing countries, where resources and capacity are limited. Our findings suggest that the Gambia is yet to achieve full implementation and benefit of the FCTC.

The Gambia has made modest progress in incorporating the FCTC into the existing national tobacco legislation in particular; the recent 2016 Tobacco Control Act is a step in the right direction. However, progress with implementation and enforcement of existing legislation since FCTC ratification has been slow. For the goals and objectives of the Treaty to be achieved, good coordination and adequate resources are needed.^14^ One example of the success and impact of a well-coordinated intervention in the Gambia was the development of a 3-year policy to increase tobacco taxation. This intervention resulted in a decrease in tobacco product importation and growth in tax revenue collection.^15^ However, more needs to be done as outlined by most of our participants; tobacco products are still very cheap and affordable. In addition, due to the geographical features of the country, increasing tobacco tax, corruption, lower penalties, and inadequate customs and border controls, most participants were concerned that this can potentially create financial incentives for cross-border smuggling and illicit trade of tobacco products. It is thus important to work with neighboring countries to help ensure that measures that prevent illicit tobacco trade and smuggling are put in place. It is further recommended that Government agencies refrain from seeking or receiving sponsorship for their programs from tobacco product importers or manufacturers from abroad.

It is also important to note that the key to controlling tobacco smuggling is not to cut tobacco taxes; it is an issue of enforcement, controlling the tobacco manufacturing industry and supporting the implementation of the WHO FCTC and the Illicit Trade Protocol.^16^

In contrast, most participants acknowledged that the Public Smoking Act (which comprehensively banned smoking in all public places), which was developed almost a decade before ratification of the FCTC, has still not been effective in protecting people from exposure to secondhand smoke. There is no safe level of exposure to tobacco smoke and the FCTCs call on parties to strive to provide universal protection within 5 years of the Treaty entering into force.^17,18^ Smoking by law enforcers, weak enforcement and low fines for violators, lack of awareness of the public smoking law among the public and enforcement authorities, and societal cultural norms are some of the barriers hindering effective implementation of smoke-free polices in the Gambia.

As previously noted by the needs assessment report by the convention Secretariat, the Gambia has had weak legislation and administrative measures to prevent access of tobacco products to minors. Most cigarettes are sold in packs of 20, but packs of 10 are also available and sale of single sticks is very common.^5^ Furthermore, participants highlighted that it is a common practice for younger people to run errands for parents or other adults, including purchasing tobacco products. We reported previously that nearly half of young people (most of them less than 16 years) were sent to buy cigarettes for their parents or others and age was not a common barrier to purchase cigarettes for half of smokers.^19^ Measures to reduce direct access of minors to cigarettes, such as requesting identification if age is in doubt, and prohibition of the sale of single cigarettes or small cigarette packs are required.

Furthermore mass-media campaigns have been shown to be effective in raising public awareness, increasing smoking cessation, and reducing smoking prevalence and uptake.^20,21^ Therefore well-designed tobacco control mass-media campaigns targeting key groups such as young people, family members, and smokers are urgently needed to improve public awareness of the adverse health, economic, and environmental consequences of tobacco consumption. Furthermore, the Ministry of Basic and Secondary Education should introduce tobacco awareness and education programs in schools. In addition, raising awareness of health workers, media professionals, decision makers, and educators have a crucial role to play in reducing current smoking prevalence, uptake, and access. Raising their awareness can potentially increase the awareness of the harmful effects of tobacco use.

Effective health warnings and labeling as outlined by the FCTC article 11 guidelines are one of the ways that can be used to increase quit attempt and reduce tobacco consumption. Moreover, graphic warnings are specifically effective in communicating harmful health effects among young people and among populations with high illiteracy rates.^11,22^ However, according to the 2017 WHO report on the global tobacco epidemic, only eight African countries have full warning label policy (large warning with all appropriate characteristics);^20^ the Gambia is not one of them. Due to high illiteracy rates and the high proportion of young people in the population, it is important for the Gambia to adopt and implement graphic health warnings.

The FCTC has raised tobacco use as a public health concern and has laid the foundation for a set of guidelines for tobacco control, which countries can adopt based on their own unique situation. However, this cannot be achieved without adequate resources.^14^ Like in many other sub-Saharan African countries, inadequate resources and inadequate capacity for tobacco control are also obstacles in the Gambia. In the midst of the need for resources for other crucial and pressing heath conditions such as infectious diseases, it can be challenging to allocate adequate resources, build capacity, and maintain tobacco control as an urgent public health priority. However, the Gambia has embarked on tobacco tax increase for several years now,^15^ and further increases and tobacco tax hypothecation can ensure some funding is available for tobacco control. For example, tobacco tax hypothecation has led to additional reduction in tobacco use in several states in the United States that use tobacco revenues to fund tobacco control programs, which support mass-media campaigns, cessation services, and policy implementation.^23–25^ Furthermore, there is evidence that smokers support higher tobacco tax if revenues are used for smoking prevention and cessation services,^26^ and there is greater public support for these approaches if the tax is earmarked for health care for combating tobacco or drug-related harms.^26,27^

## Conclusion

Our findings highlight several challenges in implementing the FCTC requirements in the Gambia; these include lack of awareness of existing polices, weak enforcement and implementation, inadequate public awareness of the harmful effects of smoking, sales to and by minors, limited resources and capacity, and inadequate research. The Gambia urgently needs to step up efforts that will help to accomplish the obligations of the FCTC. In order to achieve this, the Gambia needs to accelerate the implementation of the following measures: ensure that existing policies are fully compliant with the FCTC requirements, prioritize the enforcement and implementation of existing legislation, develop specific youth tobacco control polices and interventions and public awareness interventions (media campaigns), establish cessation services, mobilize and allocate adequate resources for tobacco control and capacity building, prevent tobacco industry interference, and strengthen surveillance and research to inform policy.

**What This Article Adds**

- The WHO FCTC presents a unique opportunity to reduce the global burden of tobacco-related mortality and morbidity.
- The FCTC is particularly important in developing countries, such as those in sub-Saharan Africa, where tobacco smoking is on the rise.
- Although several pieces of tobacco control legislation exist, enforcement and implementation of the FCTC requirements remain a major challenge in the Gambia.
- There is the need to step up efforts to implement effective tobacco control policies that are fully compliant with the FCTC requirements.
- The Gambia should also prioritize the enforcement of existing legislation, mobilize resources for tobacco control, and conduct more research to inform policy.

## Funding

This work was supported by the Medical Research Council [grant number MR/K023195/1] through the UK Centre for Tobacco and Alcohol Studies; and a scholarship funding from the Islamic Development Bank (IDB).

## Declaration of Interests

None declared.

Provenance and Peer Review

Externally peer reviewed.

Data Sharing Statement

No additional data are available.

## Supplementary Material

ntz003_suppl_Suplemmentary_File_1

ntz003_suppl_Suplemmentary_File_2

ntz003_suppl_Suplemmentary_File_3
