# Supplementary material for: Exploration of Policy Makers’ Views on the Implementation of the Framework Convention on Tobacco Control in the Gambia: A Qualitative Study
Source: Nicotine Tob Res. 2019 Jan 9;21(12):1652–9. doi: 10.1093/ntr/ntz003 (PMC6861828; doi:10.1093/ntr/ntz003)
Supplement: ntz003_suppl_Suplemmentary_File_1 [file ntz003_suppl_suplemmentary_file_1.docx]

**Supplementary file 1: Brief summary and overview of the FCTC articles**

| Article | Description | Specific articles | |
| --- | --- | --- | --- |
| 1-2 | Use of terms and relationship between the convention and parties; and legal instruments | Article 1 | Use of terms |
|  |  | Article 2 | Relationship between this Convention and other agreements and legal instruments |
| 3-5 | objectives, guiding principles and general obligations engendered by the treaty; | Article 3 | Objective |
|  |  | Article 4 | Guiding principles |
|  |  | Article 5 | General obligations |
| 6-14 | Measures relating to the reduction of demand for tobacco | Article 6 | Price and tax measures to reduce the demand for tobacco |
|  |  | Article 7 | Non-price measures to reduce the demand for tobacco |
|  |  | Article 8 | Protection from exposure to tobacco smoke |
|  |  | Article 9 | Regulation of the contents of tobacco products |
|  |  | Article 10 | Regulation of tobacco product disclosures |
|  |  | Article 11 | Packaging and labelling of tobacco products |
|  |  | Article 12 | Education, communication, training and public awareness |
|  |  | Article 13 | Tobacco advertising, promotion and sponsorship |
|  |  | Article 14 | Demand reduction measures concerning tobacco dependence and cessation |
| 15-17 | Measures relating to the reduction of the supply of tobacco | Article 15 | Illicit trade in tobacco products |
|  |  | Article 16 | Sales to and by minors |
|  |  | Article 17 | Provision of support for economically viable alternative activities |
| 18 | Protection of the environment | Article 18 | Protection of the environment and the health of persons |
| 19 | Questions related to liability | Article 19 | Liability |
| 20-22 | Scientific and technical cooperation and communication of information | Article 20 | Research, surveillance and exchange of information |
|  |  | Article 21 | Reporting and exchange of information |
|  |  | Article 22 | Cooperation in the scientific, technical, and legal fields and provision of related expertise |
| 23-26 | Institutional arrangements and financial resources | Article 23 | Conference of the Parties |
|  |  | Article 24 | Secretariat |
|  |  | Article 25 | Relations between the Conference of the Parties and intergovernmental organizations |
|  |  | Article 26 | Financial resources |
| 27 | Settlement of disputes | Article 27 | Settlement of disputes |
| 28-29 | Development of the convention | Article 28 | Amendments to this Convention |
|  |  | Article 29 | Adoption and amendment of annexes to this Convention |
| 30-38 | Final provisions | Article 30 | Reservations |
|  |  | Article 31 | Withdrawal |
|  |  | Article 32 | Right to vote |
|  |  | Article 33 | Protocol |
|  |  | Article 34 | Signature |
|  |  | Article 35 | Ratification, acceptance, approval, formal confirmation or accession |
|  |  | Article 36 | Entry into force |
|  |  | Article 37 | Depositary |
|  |  | Article 38 | Authentic texts |
