# Supplementary material for: Exploration of Policy Makers’ Views on the Implementation of the Framework Convention on Tobacco Control in the Gambia: A Qualitative Study
Source: Nicotine Tob Res. 2019 Jan 9;21(12):1652–9. doi: 10.1093/ntr/ntz003 (PMC6861828; doi:10.1093/ntr/ntz003)
Supplement: ntz003_suppl_Suplemmentary_File_2 [file ntz003_suppl_suplemmentary_file_2.docx]

**Supplementary file 2: Key Policy maker’s interview guide**

The aim of the project is to provide detailed insight into tobacco use, and to assess barriers to implementation of tobacco policies in The Gambia. You taking part in this research study will help us to identify the extent to which the FCTC has been implemented and barriers to its effective implementation in The Gambia. We have the pleasure to invite you to take part in this research study. Check if participant has signed consent, if they have any questions before starting the interview.

**Part 1: Introduction**

1. How long have you been a member of this committee and what’s your role?
2. What do you think about the prevalence of tobacco use in The Gambia?

- Do you think prevalence is different in youth and adults?
- How about among males and females

**Part 2: Tobacco Polices and FCTC**

1. Can you tell me about any policies you are aware of for controlling the tobacco epidemic in Gambia?
2. How effective do you think these polices are in controlling the epidemic?

Now moving to the WHO Framework Convention on Tobacco Control (FCTC). Note: Give a brief information of the FCTC for those who did not mention it among the existing policies)

1. Tell me what do you know about the FCTC
2. Do you think the tobacco polices in The Gambia has adequately covered the FCTC requirement?
3. Which areas do you think progress has been made in a bid to implement the FCTC?
4. Are there other specific areas that have been carried out by The Gambia as a unique strategy

Part 3: Achievements and challenges:

1. Achievements

Can you describe the existing tobacco policies with regard to achievements in relation to the following specific areas?

1. Price tax measures to reduce demand on tobacco products
2. Protection from exposure to tobacco smoke (smoke free policy)
3. Regulation of the contents and disclosure of tobacco products
4. Packaging and labelling of tobacco products
5. Education, communication, training and public awareness (media campaigns)
6. Demand reduction measures concerning tobacco dependence and cessation services and provision of support for quitting
7. Illicit trade in tobacco products
8. Sales to and by minors
9. Research and surveillance
10. Challenges
11. Now with regards to some of the achievements you have mentioned above can you describe any challenges that you think still exist.

Part 4: Recommendations and way forward

1. Can you think of any recommendations on the way forward required in the area of tobacco control and policy implementation
2. What do you think the next step should be for Gambia in addressing tobacco control

- How can this be achieved
- What may be some of the barriers and challenges
- How can these be address

**Closing Remarks**

- Is there anything you would like to add that we have not already covered?
- Thank participant for their time and reassure confidentiality.

Sociodemographic details:

1. Job title/designation
2. Representing institution
3. Age
4. Gender
5. Have you ever use any tobacco products
6. Have you ever smoke cigarette
7. Do you currently smoke cigarettes

**Thank you**
