# Supplementary material for: Exploration of Policy Makers’ Views on the Implementation of the Framework Convention on Tobacco Control in the Gambia: A Qualitative Study
Source: Nicotine Tob Res. 2019 Jan 9;21(12):1652–9. doi: 10.1093/ntr/ntz003 (PMC6861828; doi:10.1093/ntr/ntz003)
Supplement: ntz003_suppl_Suplemmentary_File_3 [file ntz003_suppl_suplemmentary_file_3.docx]

**Supplementary file 3: The procedure and steps of the framework method of analysis conducted in NVivo**

| Steps | Activities in NVivo | Comments |
| --- | --- | --- |
| 1 | Importation of transcript and setting each transcript in to a case node | All transcripts were imported as case node that represent each participant |
| 2 | Coding | The coding of the first few transcripts |
| 3 | Creation of an analytical framework or thematic node hierarchy | A note hierarchy of the themes and sub-themes identify during the coding of the first few transcript was set up |
| 4 | Applying the analytical framework | All transcripts were coded to the theme nodes hierarchy |
| 5 | Charting data in to the frame matrix | A framework matrices was created from the case node (as rows) and the theme nodes (as columns). The intersection of each case and thematic node forms matrix cell, and this is where code (source content) that relates to the case and theme is found |
| 6 | Mapping and interpreting the data | A Summarized framework matrices was created that links to the themes, cases and the supporting source materials. |

An illustrative sample of the framework matrix used in Nvivo during analysis


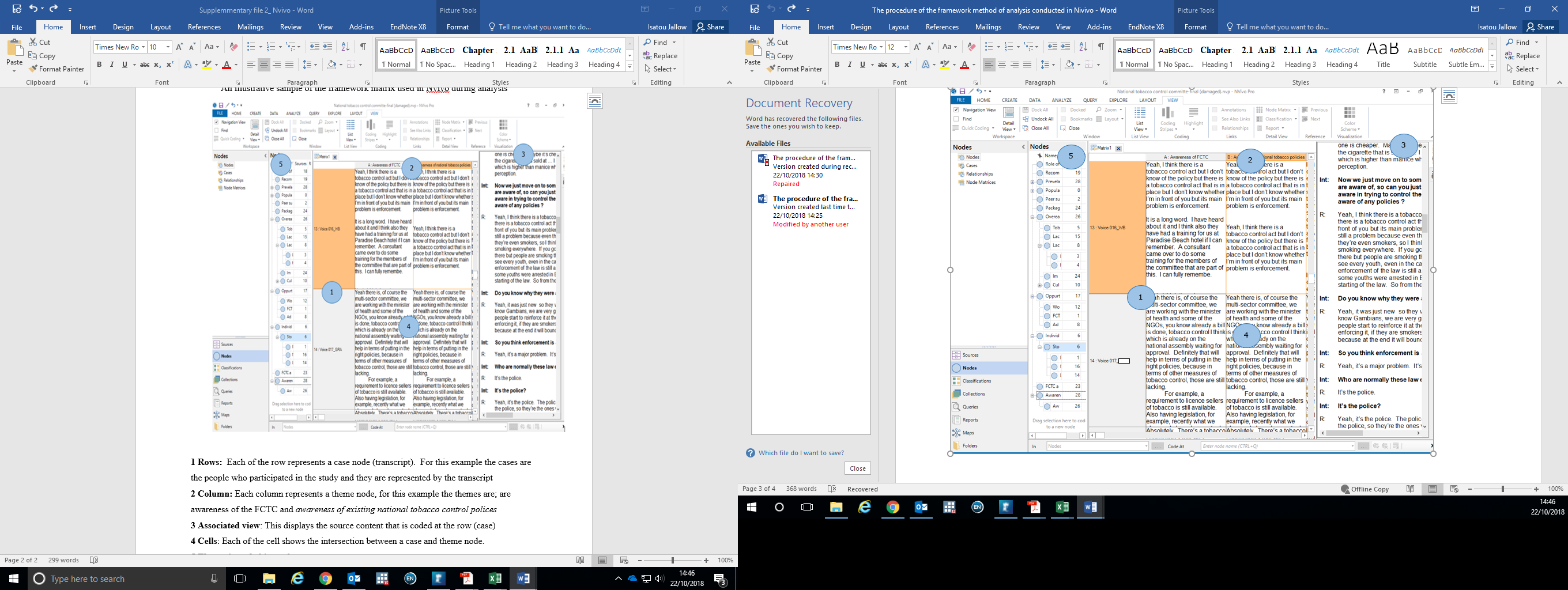


**1** **Rows:** Each of the row represents a case node (transcript). For this example the cases are the people who participated in the study and they are represented by the transcript

**2** **Column:** Each column represents a theme node, for this example the themes are; are awareness of the FCTC and *awareness of existing national tobacco control polices*

**3** **Associated view**: This displays the source content that is coded at the row (case)

**4** **Cells**: Each of the cell shows the intersection between a case and theme node.

**5** **Thematic node hierarchy:** Display of the themes and sub-themes map in a chart
